# Foreign bodies in the aerodigestive tract: time for comprehensive preventive measures

**DOI:** 10.4314/ahs.v22i2.42

**Published:** 2022-06

**Authors:** Peter Oladapo Adeoye, Olushola Abdulrahman Afolabi, Habeeb Kayodele Omokanye, Ifedolapo Olaoye, Oluwaseun Rukeme Akanbi, Segun Segun-Busari, Olusola Abidemi Morohunfade Adesiyun, Olufemi Adebayo Ige, Abdulrazaq Olasunkanmi Akiode, Ololade Aderinola Wuraola, Mohammed Baba Abdulkadir, Joshua Olayinka Oni

**Affiliations:** 1 Division of Thoracic & Cardiovascular Surgery, Department of Surgery, University of Ilorin; 2 Department of Otorhinolaryngology, University of Ilorin; 3 Division of Thoracic & Cardiovascular Surgery, Department of Surgery, University of Ilorin Teaching Hospital; 4 Department of Radiology, University of Ilorin and University of Ilorin Teaching Hospital; 5 Department of Anaesthesia, University of Ilorin; 6 Department of Otorhinolaryngology, University of Ilorin Teaching Hospital; 7 Department of Paediatrics and Child Health, University of Ilorin and University of Ilorin Teaching Hospital; 8 Department of Anaesthesia, University of Ilorin Teaching Hospital

**Keywords:** Foreign body, Aspiration, Ingestion, Aerodigestive tract, Impaction

## Abstract

**Background:**

Foreign body (FB) in the aerodigestive tract presents more commonly in children and remains a surgical emergency with potential for fatal complications.

**Objectives:**

To describe management and outcomes of aerodigestive FB managed at University of Ilorin Teaching Hospital (UITH) and proffer preventive measures.

**Methods:**

A 9-year retrospective review of all patients with foreign body in the aerodigestive tract managed between March 2011 and July 2020.

**Results:**

Sixty-six patients were studied. Median age was 9years with M:F ratio =1.6:1. FB was ingested in 38(57.6%) patients, aspiration occurred in 28(42.4%). Denture was most common FB 20(30.3%); plastic whistle/valve placed in dolls or football accounted for 4(6.1%). When ingested, FB was impacted in cervical 17(44.7%), upper thoracic 10(26.3%) and middle thoracic 2(5.3%) oesophagus. Oesophagoscopy was used in 30(8.9%) for retrieval. When aspirated, FB was located in the right bronchus 10(35.7%), left bronchus 7(25.0%), hypopharynx and trachea 2(7.1%) each, and cricopharynx 1(3.5%); no FB was found in 3(10.7%) patients. Direct Laryngoscopy was the method of retrieval in 3(10.1%) patients while others had rigid bronchoscopy. Mortality rate was 1.5%.

**Conclusion:**

Children are most vulnerable group. Preventive effort should include public health education and close monitoring of children by parents and care givers during play.

## Introduction

Foreign body (FB) ingestion and aspiration are common surgical emergencies in otolaryngology and cardiothoracic practice. It is commoner in the extremes of ages but especially among the paediatric age group with a wide spectrum of clinical problems and surgical complications.^1^,^2^,^3^ Despite significant advances in imaging and endoscopic techniques, they remain a source of high morbidity and mortality worldwide.4 Whether the FB got into the aero-digestive tract accidentally or deliberately, the type of FB and the site of impaction dictate the case-management by the otolaryngologist or cardiothoracic surgeon. Surgical management of a FB in the upper aerodigestive tract (ADT) is prompt rigid-endoscopic retrieval under conditions of maximum safety and minimal trauma.^5^, ^6^

FB in the air and food passages are the sixth most common cause of accidental death in the United States with over 3,000 deaths resulting from such aspirations per year.^7^ The largest FB surveillance registry in Europe reports incidence of non-food FB amongst European Union children aged 0–14years as 50,000 per annum, and 1% are fatal. About 10,000 are inorganic and 2,000 involve toys.^8^ Kirfi et al described a prevalence of 0.61% and an average annual incidence of 0.13% over a 5-year period in a Hospital in northern Nigeria.^9^

As toddlers play, they may put FB in their mouths unnoticed by an adult observer. Any attempt to talk, laugh or sing, may result in the FB being swallowed or aspirated inadvertently. In absence of an adult witness, the classical diagnostic history of a FB aspiration may be missed.

Retained FB may be initially asymptomatic but later produce a variety of respiratory symptoms.^7^,^10^ It may predispose to intermittent tracheobronchitis or recurrent pneumonia, causing more confusion since this age group ordinarily has a high incidence of tracheobronchitis, inflammatory diseases, and asthma.^6^,^7^ Frequently, these children are wrongly treated for prolonged periods for asthma, pneumonia, or allergy.^7^,^11^ Nevertheless, unexplained recurrent pneumonia, other respiratory illnesses, or conditions that do not respond to appropriate medical management in children should always raise the suspicion of aspirated FB.^6^,^7^,^12^

This study investigates the incidence, management and complications of FB retrieval from the ADT in our setting, with a comparison of our 9 years' experience with what is described in the literature. We suggest preventive measures.

## Patients and Methods

Records of patients with clinical or radiological suspicion of FB ingestion or aspiration referred for emergency care at the otolaryngology or cardiothoracic divisions of UITH between March 2011 and July 2020 were retrospectively revised. Patients were managed independently by the units but where patients condition dictated, combined team approach was implemented. Information extracted included age, gender, type of FB, clinical-radiological presentation, endoscopic retrieval method, complications, hospital stay and outcomes. Data was complemented with information from operation notes. Permission was obtained from the Institutional Ethical Review Board with assigned number NHREC/02/05/2010 and approval number ERC PAN/2020/02/0116; and patients' identities kept confidential.

## Results

**Figure 1:**
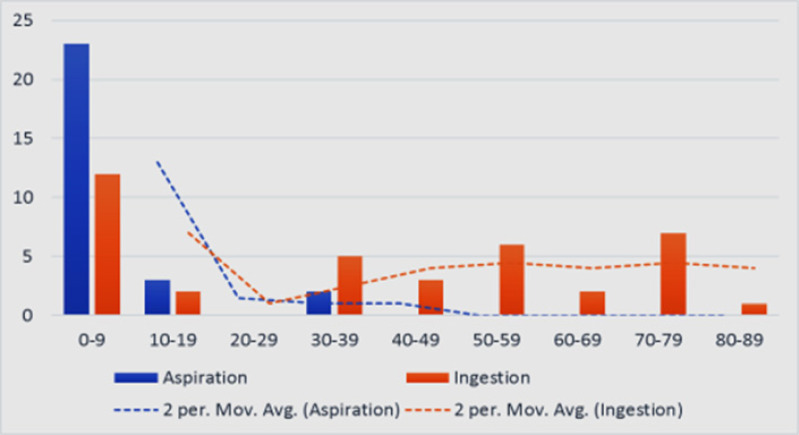
Bar chart of age grouping versus aspiration or ingestion

Sixty-six patients presented with FB in the ADT over the 9years-5months period. Forty-one (62.1%) patients were males (M:F ratio of 1.6:1). Ages ranged from 8months to 80years (median 9years). The most frequent age group was 0–9years accounting for 35(53%) followed by 30–39years and 70–79years with 7(10.6%) each (Figure 1).

Twenty-four (36.4%) were of pre-school (≤5years) while 37(56.1%) were of paediatric (≤15years) age groups. Whilst 38(57.6%) patients presented with FB ingestion, 28 (42.4%) had aspiration. Though the type of object was either not stated in 9(13.6%), none seen in 3(4.5%) and not applicable in a patient with suspicious history and retropharyngeal abscess who declined intervention 1(1.5%), denture accounted for the vast majority of FBs being present in 20(30.3%) patients. Metallic bolt/screws/cap, disc-lithium watch battery and plastic whistle/valve placed in dolls or football accounted for 4(6.1%) each; peanut – 3(4.5%); Light Emission Diode (LED) bulb, ear ring, biro base cork, office pin and kola-nut – 2(3.0%) each. Others, accounting for 1(1.5%) each include vegetable fragments, coin, plastic bead, maize, sharpener, metallic washer, sugarcane peel and key. Out of the 53 patients whose objects were identified, 45(84.9%) were of inorganic material while 8(15.1%) were organic (Table 1).

**Table 1:** Frequency of foreign body aspirated or ingested, classified into inorganic and organic types

Classification	Type	Frequency		Total
		Aspiration(%)	Ingestion(%)	
	Denture		20(100)	20
	Metallic bolt/screw/cap	3(75)	1(25)	4
	Disc-lithium watch battery		4(100)	4
	LED bulb	2(100)		2
**Inorganic (45)**	Ear ring	2(100)		2
	Biro base cork	2(100)		2
	Office pin	2(100)		2
	Plastic whistle/ball/doll valve	4(100)		4
	Coin		1(100)	1
	Plastic bead	1(100)		1
	Sharpener		1(100)	1
	Metallic washer		1(100)	1
	Key		1(100)	1
**Organic (8)**	Kolanut	1(50)	1(50)	2
	Sugarcane peel	1(100)		1
	Peanut	3(100)		3
	Vegetable	1(100)		1
	Maize	1(100)		1
**Not stated**		2 (22.2)	7(77.8)	9
**No object seen**		3(100)		3
**Not applicable**		0	1(100)	1
**TOTAL**		**28(42.4)**	**38(57.6)**	**66**

Though both aspiration and ingestion of FBs were more common among children and young adults, no occurrence of aspiration occurred in the older adults nor elderly. Neither aspiration nor ingestion occurred in 20–29 age group (Figure 1). The median age for aspiration was 4.5years while for ingestion was 40years (Mann Whitney's test = 0.000). The oldest age recorded for aspiration was 39years and 80years for ingestion. When incidence of as-piration or ingestion was compared between preschool and above preschool age groups, Fisher's exact test was 0.019 and Odds ratio = 3.72 while paediatric versus adult had values of 0.000 and 18.06 respectively (Table 2).

**Table 2:** Comparison of Preschool to above preschool age groups and paediatric to adult patients in the likelihood of aspiration or ingestion of foreign body

Categories	Group	Aspiration(%)	Ingestion(%)
	≤5	15(53.6)	9(23.7)
**<5** **vs >5years**	>5	13(46.4)	29(76.3)
	Total	28(100)	38(100)
	Fisher's exact and Mann Whitney's tests		0.019
	Odds ratio		3.72
**<15** **vs >15years**	≤15	25(89.3)	12(33.3)
	>15	3(10.7)	26(89.7)
	Total	28(100)	38(100)
	Fisher's exact and Mann Whitney's tests		0.000
	Odds ratio		18.06

Gender comparison for aspiration and ingestion, with 16 males of 28 cases of aspiration (57.1%) and 25 males in 38 cases of ingestion (65.8%) had P = 1. We had 2(3.3%) morbidities and 1(1.5%) mortality.

**Figure 2:**
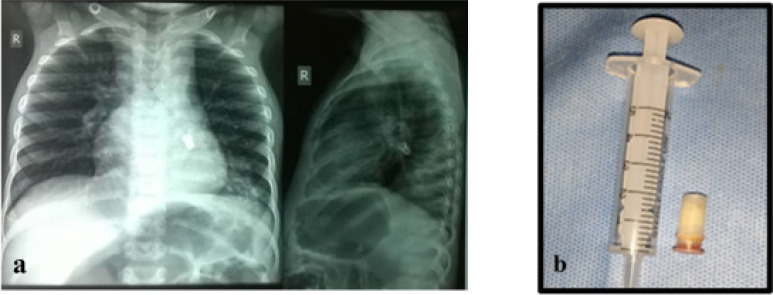
a) AP and lateral CXR shows whistle-valve in left main bronchus b) Whistle-valve retrieved

**Figure 3:**
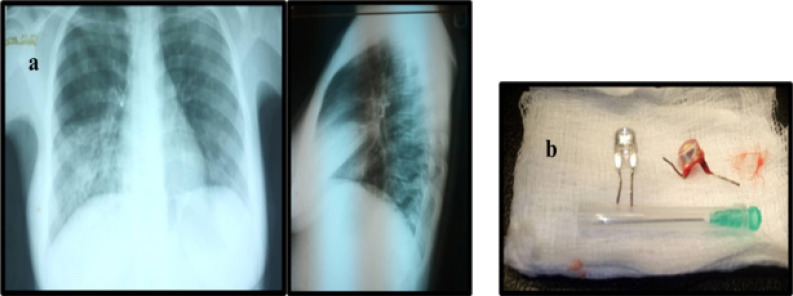
a) AP and lateral CXR – LED bulb element in right main bronchus with atelectasis of middle and lower lobes. b) LED bulb retrieved (right), prototype (left).

Most common FB aspirated of 28 cases were plastic whistle/valve in dolls or football with 4(14.3%) cases (Figure 2). Peanuts and metallic bolts/screw/cap accounted for 3(10.7%) each while LED bulb (Figure 3), ear ring and biro base cork occurred in 2(7.1%) cases each. Others had 1(3.5%) each (Table 1). Ten (35.7%) were located in the right bronchus (7-right main bronchus, 3-bronchus intermedius), 7(25.0%) were in the left bronchus (4-left main bronchus, 3-lower lobe bronchus), 2(7.1%) were in both bronchial trees (peanut fragments and vegetables). Another 2(7.1%) was located in the hypopharynx and trachea respectively while 1(3.5%) was at the cricopharynx and no object was found in 3 (10.7%). The location was not stated in 1 patient (P = 0.003). Only 3(10.7%) had their FB retrieved by direct laryngoscopy (2 located at the hypopharynx, 1 at cricopharynx) all others had rigid bronchoscopy (Table 3).

**Table 3:** Foreign body retrieval procedure.

Presentation	Procedure	Frequency	Percentage
**Aspiration**	Rigid bronchoscopy	25	89.3
	Laryngoscopy	3	10.7
	**TOTAL**	28	100

**Ingestion**	Rigid oesophagoscopy only	30	78.9
	Oesophagoscopy + Oesophagotomy	3	7.9
	Oesophagoscopy + Gastrotomy	1	2.6
	Oesophagotomy only	1	2.6
	Nil – Patients declined procedure/FB migrated into intestine	3	7.9
	**TOTAL**	38	100

Amongst 38 cases of FB ingestion, 20(52.6%) were secondary to dentures, 4(10.5%) were disc-watch batteries while others were 1(2.6%) each (Table 2). The most frequent site of impaction was in the cervical oesophagus – 17(44.7%). There were 10(26.3%) and 2(5.3%) in the upper and mid thoracic oesophagus respectively. None was located in the lower oesophagus (Table 4).

**Table 4:** Location of foreign bodies ingested

FB type	Location of Object					Total
	Cervical(%)	Upper thoracic (%)	Mid thoracic (%)	Not applicable (%)	Not stated(%)	
**Denture**	13(65)	4(20)	2(10)	1(5)		20
**Disc-watch** **battery**	3(75)	1(25)				4
**Kolanut**		1 (100)				1
**Metallic** **bolt/screw**				1(100)		1
**Coin**		1(100)				1
**Sharpener**		1(100)				1
**Metallic** **washer**	1(100)					1
**Key**		1(100)				1
**Not** **stated**		1(14.3)			6(85.7)	7
**Not** **applicable**				1(100)		1
**Total**	**17(44.7)**	**10(26.3)**	**2(5.3)**	**3(7.9)**	**6(15.8)**	**38**

Rigid oesophagoscopy (RO) was utilized in 30(78.9%) of cases. Two patients refused oesophagoscopic evaluation despite history of FB ingestion and in 1 patient, the FB had descended into the bowel (Table 3). Five patients had operative removal. An 8-month-old girl who ingested a 3-volt disc battery which migrated distally into the stomach during RO had immediate mini laparotomy and gastrotomy. Three patients, a 74-year-old male, and two females 50 and 52 years old had failed attempt at RO removal necessitating left neck (for the male and 50-year-old female) and right neck (for the 52-year-old female) exploration with oesophagotomy. The females had sustained oesophageal lacerations on posterior and right lateral wall respectively form sharp-ragged edge of the denture while applying traction at RO. Another 74-year-old man had elective right postero-lateral thoracotomy and oesophagotomy because of the more distal location of denture in mid-thoracic oesophagus and chronicity of impaction (>6 months) with fibrosis.

## Discussion

Toddlers and younger children are more likely to place objects in the mouth during play. Thirty-seven (56.1%) of the 66 patients in our review were paediatric patients with a male preponderance similar to earlier studies^9^,^13^ including reports by Odelowo^14^, and Alabi et al.^15^ from our institution. Under five children have underdeveloped gag reflex with impaired neuromuscular coordination of the pharynx and larynx^16^, hence higher tendency to aspirate than ingest accidentally compared with adults. This study recorded more aspirated FB among the under-fives than ingested FB. (Table 2)

Ear-rings, biro base-cork, peanuts and LED bulb were the most frequently aspirated FBs. The commonest side of the airway affected was the right.^6^ As previously documented, this is due to peculiar anatomic disposition of right main bronchus which is usually short and wider than the left, with a lesser angle of deviation from the tracheal axis.^6^

Denture was the commonest FB in this series, all occurring as ingestion (Table 1). This may be explained by general increase in longevity and heightened cosmetic awareness among Nigerian elites, many of who use dentures for cosmetic reasons.^17^ Large size of dentures and non-expansible nature of the cartilaginous wall of the laryngotracheal lumen make it impossible for dentures to track into the airway. Similarly, its irregular shape prevents migration deep into the oesophagus. Hence, cervical and proximal thoracic oesophagus were common location for its impaction in this study.

We observed an increasing incidence of disc-battery ingestion, perhaps due to increase usage in small electronic devices and toys.^6^ This is at variance with earlier studies where coin was the leading FB ingested.^15^ In contemporary Nigeria, unlike the experience by Odelowo two decades ago^14^, coins are no longer commonly utilized in business/economic transactions and this may account for its low incidence in this study.

**Figure 4:**
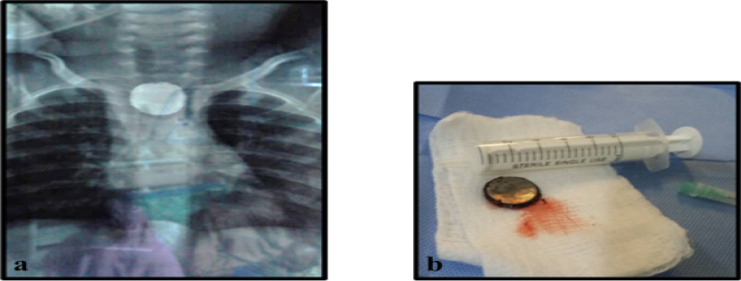
a) AP CXR - round radio-opaque object in proximal oesophagus. b) Disc-battery retrieved

Disc battery ingestion should be managed with utmost urgency (figure 4) because of potential risk of oesophageal injury by indirect corrosive action, low voltage burns and pressure necrosis.^6^ Long term implication may cause acquired tracheoesophageal fistula.^18^ The battery migrated into the stomach during oesophagoscopy in one patient, necessitating laparotomy and gastrotomy.

We recorded no FB ingestion or aspiration between ages 20–29 years. This is the age group for self-awareness and self-care. Dentition is intact hence they hardly wear dentures; even if they do, it is well-fitting. Furthermore, unlike the very young and elderly, laryngeal reflex, laryngoesophageal coordination and oesophageal peristalsis are intact.

Endoscopic removal is preceded by imaging to determine the location of FB, evaluate surrounding tissue and exclude complications like migration, viscus perforation or tissue penetration. Preoperative imaging thus facilitates planning and increases chances of successful removal. Radiolucent FB not visible on plain radiograph may be displayed on fluoroscopy, CT-scan or MRI. Ventilation-perfusion scan may be helpful in aspirated FB.^12^

FB in the oesophagus that defies removal by oesophagoscopy would require oesophagotomy.^6^ Similarly, while laryngoscopy is indicated in FBs located above the level of cricoid cartilage, bronchoscopy is utilized for FBs lodged in the tracheobronchial lumen. However, where bronchoscopy fails, external approach by bronchotomy via thoracotomy is used. Three of our patients (7.9%) had neck exploration for impacted denture in cervical oesophagus after failed RO. Another one, chronically impacted in mid-oesophagus had exploratory thoracotomy and oesophagotomy (without prior oesophagoscopy). This may have been approached via video-assisted thoracoscopy (VATS) where the facility is available.^18^ Thirty (78.9%) others had successful retrieval by rigid oesophagoscopy alone (Table 3). Though flexible oesophagoscopy (FO) is being advocated as having theoretical advantage over RO as it is performed under local anaesthesia, less discomforting to the patients and lower complication rates; the optimal exposure provided by the wider lumen of RO facilitates retrieval of large and sharp-pointed objects. Also, children will not tolerate local anaesthesia for use for FO.^19^ Use of FO in our institution is limited to diagnostic purpose.

Mortality rate of 3.6% recorded from aspirated FB is high compared with 1% documented in earlier studies.^6^,^21^,^22^ That was one of the 3 patients who had perioperative cardiac arrest during bronchoscopy. Ajiya et al in Nigeria reported a 22.9% complication rate for endoscopic retrieval of FB in the ADT.^23^

We conclude that aerodigestive foreign bodies remain a frequent indication for emergency oesophagoscopy and bronchoscopy. Children are most vulnerable and are particularly prone to aspiration. Increased awareness and availability of orthodontist services has increased the use of dentures in adults yet not matched by adequate maintenance of the device has increased its occurrence as an ingested and impacted agent.

Therefore, the need for preventive measures cannot be overemphasized given the high risk of mortality associated with FB in the ADT in spite of advances in imaging and endoscopic technology worldwide. A nationwide educational campaign in Israel achieved 35% reduction in incidence of FB aspiration in children over a 3-year period.24 We therefore, recommend education of parents, care givers, school teachers, pupils and the entire community on the danger of FB ingestion or aspiration and appropriate preventive measures.

Teaching on preventive measures should be included in health education of mothers at the antenatal clinic and in curriculum for school pupils. Writing materials especially biros with detachable base should be prohibited from use in primary and secondary schools while recommending those with non-removable base. Dentures should be fabricated with radio-opaque component to ease identification and routine follow-up to the orthodontist should be encouraged for early identification and rectification of faulty fitting.

Our study is limited by its retrospective design which was responsible for some missing information and also by lack of data on long term follow-up of the patients.
